# Genome Sequence of a Gammaherpesvirus from a Common Bottlenose Dolphin (*Tursiops truncatus*)

**DOI:** 10.1128/genomeA.00777-17

**Published:** 2017-08-03

**Authors:** Andrew J. Davison, Kuttichantran Subramaniam, Karen Kerr, Jessica M. Jacob, Nelmarie Landrau-Giovannetti, Michael T. Walsh, Randall S. Wells, Thomas B. Waltzek

**Affiliations:** a MRC-University of Glasgow Centre for Virus Research, Glasgow, United Kingdom; b Department of Infectious Diseases and Pathology, College of Veterinary Medicine, University of Florida, Gainesville, Florida, USA; c Department of Large Animal Clinical Sciences, College of Veterinary Medicine, University of Florida, Gainesville, Florida, USA; d Chicago Zoological Society’s Sarasota Dolphin Research Program, c/o Mote Marine Laboratory, Sarasota, Florida, USA

## Abstract

A herpesvirus genome was sequenced directly from a biopsy specimen of a rectal lesion from a female common bottlenose dolphin. This genome sequence comprises a unique region (161,235 bp) flanked by multiple copies of a terminal repeat (4,431 bp) and contains 72 putative genes. The virus was named common bottlenose dolphin gammaherpesvirus 1.

## GENOME ANNOUNCEMENT

Health assessments of common bottlenose dolphins (*Tursiops truncatus*) in Sarasota Bay, FL, were carried out in 2015 (1). Physical examination of a 13-year-old long-term resident female on 15 May determined that the animal weighed 142.2 kg, was lactating, had mild lung disease, and exhibited a raised proliferative cutaneous lesion at the border of the rectum. A biopsy specimen of the lesion was taken and stored at −80°C. DNA was extracted by using a Qiagen DNeasy blood and tissue kit. A sequencing library was prepared by using an Illumina Nextera XT DNA library preparation kit and sequenced by using a 600-cycle version 3 cartridge on an Illumina MiSeq.

The data set of 8,994,640 quality-trimmed reads was assembled *de novo* by using SPAdes (2). A large herpesvirus-related contig was extended and joined to other contigs manually, essentially forming a circular sequence consisting of a unique region (U) linked to a complete and a partial copy of a terminal repeat (TR). The right genome terminus was mapped to a nucleotide in TR from the presence of multiple reads commencing at the same position, and the adjacent nucleotide was assigned as the left genome terminus. The sequence contained seven large tandem repeats that were possibly heterogeneous and could not be solved by additional sequencing of PCR products. Representative solutions consistent with the size of PCR products and the available sequence data were adopted. Coverage analysis indicated that the linear viral genome consists of U flanked on each side by multiple copies of TR. The final sequence was represented as U followed by a complete and a partial copy of TR. The size of this sequence was 167,212 bp, with the sizes of U and TR being 161,235 bp and 4,431 bp, respectively. The integrity of the sequence was assessed by inspection of a read assembly generated by using Bowtie 2 (3). A total of 626,924 reads (7.0% of the total) aligned at an average coverage of 1,092 reads per nucleotide.

Predicted amino acid sequences from open reading frames (ORFs) of >50 codons were screened for relatives in other organisms (especially herpesviruses) and to locate probable initiation codons and splicing patterns. Substantially overlapping ORFs and ORFs of <150 codons that lacked relatives and other features (e.g., hydrophobic domains) were ruled out. Among the final set of 72 genes, 4 (De4, De7, De9, and De10) lacked relatives and other features, and were considered marginal. Phylogenetic analyses indicated that the virus from which the genome presumably originated is a deeply rooted member of subfamily *Gammaherpesvirinae* of family *Herpesviridae*.

The virus was named common bottlenose dolphin gammaherpesvirus 1 strain Sarasota (proposed species *Delphinid gammaherpesvirus 1*) and is the first herpesvirus from a marine mammal for which a genome sequence has been determined. Alphaherpesviruses and gammaherpesviruses have been reported previously in the cetacean families Balaenopteridae, Delphinidae, Kogiidae, Monodontidae, Phocoenidae, Physeteridae, and Ziphiidae (4–8).

### Accession number(s).

The common bottlenose dolphin gammaherpesvirus 1 strain Sarasota genome sequence has been deposited in GenBank under accession no. KY965444.
